# Immunotherapy of small cell lung cancer based on prognostic nutritional index

**DOI:** 10.3389/fimmu.2025.1560241

**Published:** 2025-05-26

**Authors:** Xinling Zhang, Yaxuan Zhang, Jiaqi Zhang, Jinghua Yuan, Shuying Zhu, Xiaoping Li

**Affiliations:** ^1^ Key Laboratory of Artificial Organs and Computational Medicine in Zhejiang Province, Shulan International Medical College, Zhejiang Shuren University, Hangzhou, China; ^2^ Department of Microbiology Laboratory, Yiwu Center for Disease Prevention and Control, Yiwu, China

**Keywords:** small lung cancer, nutrition, immunotherapy, diet, prognostic nutritional index

## Abstract

Platinum-based first-line chemotherapy for small lung cancers has been a mainstream therapy for the past several decades. However, its efficacy has been suboptimal, and the research is now focused on improving the treatment and prognosis of competitive nutrition and multidrug combination techniques. Small cell lung cancer (SCLC) is not only affected by smoking, age, sex and other external factors, but also the tumor micro-environment and the nutritional status of patients themselves are of great significance for the prevention and treatment of SCLC, a malignant tumor. According to past research, malnutrition is related to the intolerance to immunotherapy, decline in quality of life, psychological disturbances, and low survival rates and prognosis. Numerous studies have shown that a low Prognostic Nutritional Index (PNI) serves as an independent prognostic factor linked to reduced overall survival across various cancer types. Additionally, PNI has been associated with disease-free survival and progression-free survival in certain cancers, such as lung cancer (LC). Recent research has indicated that the PNI can serve as an independent predictor of both long-term outcomes and short-term complications in SCLC patients. However, a systematic consensus on this matter has yet to be established. This paper focuses on the role and influence of PNI in the immunotherapy of SCLC, and proposes the possibility of dietary therapy for SCLC patients under the guidance of PNI. Finally, the authors pointed out that PNI will become a new strategy for comprehensive immunotherapy of SCLC.

## 1 Introduction

### 1.1 Small cell lung cancer

Small cell lung cancer (SCLC) is the most common aggressive, poorly differentiated, high-grade neuroendocrine carcinoma, accounting for about 10%-15% of lung cancer (LC) (1). Its typical clinical manifestations are fatigue, appetite loss, weight loss, dyspnea, hemoptysis and chest pain, which lead to the decline of physical function and quality of life (2). With the increase of life and work pressure and the emergence of poor eating behaviors, the incidence of SCLC is increasing year by year worldwide. SCLC is usually fatal, with a 5-year survival rate of 7% (3). Significantly, even though the proportion of SCLC cases has declined over the years, the overall survival rate for patients is still notably low. SCLC accounts for an estimated 250,000 new cases worldwide each year and contributes to at least 200,000 deaths (4). Currently, the clinical progress of SCLC is very slow, so people pay more and more attention to SCLC. While SCLC initially shows a strong response to platinum-based chemotherapy, it is prone to relapse and eventually universal drug resistance (5). SCLC is often refractory to second-line therapy (6). Over the past decade, immunotherapy has revolutionized the initial treatment of SCLC and has led to significant enhancements in survival rates and clinical results (7).

### 1.2 Definition and clinical significance of prognostic nutritional index

The Prognostic Nutritional Index (PNI) was initially proposed by Buzby and subsequently refined by the Japanese scholar Onodera (8, 9). It serves as a nutritional assessment tool and predictor of surgical risk, originally designed to evaluate perioperative conditions and predict surgical risks in patients with gastrointestinal tumors (10).

PNI is a concise index comprising only two parameters: serum albumin (ALB) and total lymphocyte count (TLC). ALB, synthesized by the liver, is a critical component of plasma that maintains colloid osmotic pressure. Chronic inadequate protein intake can lead to decreased levels of ALB, making it an indicator of chronic protein malnutrition and reflecting overall nutritional status. Garth et al. (11) reported that ALB can predict the length of hospital stay in gastric cancer patients. TLC serves not only as a nutritional index but also as an immune index, reflecting immune function. Factors such as age, malnutrition, and immune dysfunction can result in decreased TLC levels, which are associated with patient morbidity and mortality (12, 13). The formula for calculating PNI is:


$$\text{PNI}=\text{ALB }(\text{g}/\text{L})+5\times \text{TLC }(10^{9}/\text{L})$$

A PNI score greater than 50 indicates normal nutritional status. Scores between 45 and 50 suggest mild malnutrition, yet gastrointestinal resection and anastomosis remain safe. Scores between 40 and 45 indicate significant malnutrition, posing risks for gastrointestinal surgery. Scores below 40 signify severe malnutrition, rendering the patient unsuitable for surgery. Initially, Onodera used PNI exclusively for perioperative assessment and surgical risk prediction in gastrointestinal patients. However, recent studies by Nozoe et al. have shown that PNI can also assess the aggressiveness of malignancies (14, 15).

### 1.3 Immunosuppressive checkpoint therapy

Immunosuppressive checkpoint therapy has made landmark progress in the treatment of patients with SCLC, and has been approved as first-line and third-line treatment options for extensive or relapsed SCLC (16). These include antibodies targeting cytotoxic T-lymphocyte antigen-4 (CTLA-4), programmed death checkpoint-1 (PD-1), and programmed death ligand-1 (PD-L1) to block immune regulatory checkpoints on tumor cells, immune cells, fibroblasts, and endothelial cells (17). However, these models do not consistently demonstrate adequate predictive validity. In conclusion, patients diagnosed with LC who have low PD-L1 expression may benefit from immunotherapy. Alternatively, individuals with increased PD-L1 expression might not respond well to immunotherapy, and the practical use of these indicators could be constrained by the high costs of testing and the diverse nature of tumor samples. (18).

Interestingly, the combination of immunotherapy, chemotherapy, radiotherapy and targeted therapy can achieve greater therapeutic effect (19). Several earlier studies have shown that polyphenolic phytochemicals in plant foods can prevent multiple forms of cancer (20, 21). Changes in dietary habits may profoundly affect the occurrence and development of cancer. Consequently, this might be a new and affordable combination immunotherapy strategy to reduce the increasing cancer burden (22).

## 2 The role of changes in PNI in cancer treatment

SCLC is an invasive neuroendocrine tumor. The prognosis for patients with this disease is very poor because of its rapid cell proliferation, high growth rate, and early-stage metastasis (23). Over the last thirty years, treatment plans for SCLC patients have been quite restricted, with few new options available (23). In the 1970s and 1990s, surgical resection was usually the main treatment for SCLC. Because the results of surgery-based randomized clinical trials have not been favorable, this method was usually not adopted (24). Due to the limitations of surgical treatment, systemic chemotherapy needs to be combined to improve the therapeutic effect. Since 1985, the combined treatment of cisplatin and etoposide has been the preferred chemotherapy regimen for patients with SCLC (23, 25). Chemotherapy can prolong the survival period of patients to a certain extent, but its toxicity and side effects still cannot be ignored. As cancer research progresses, the US Food and Drug Administration in 2018 approved the use of atezolizumab alongside first-line platinum-based dual-drug chemotherapy for treating various stages of the disease. This approval establishes immune checkpoint blockade as a new treatment approach for SCLC, which is of great significance (26). Since then, immunotherapy has gradually become the recommended treatment method for SCLC due to its remarkable efficacy and few side effects.

Many studies have shown that PNI is related to the survival rate of various cancers and can reflect the chronic inflammation, immune status and nutritional status of cancer patients. Zhang et al. conducted a study on the changes of the PNI index in patients with gastrointestinal cancers receiving immunotherapy. Through statistical analysis, it was found that an increase in the PNI index could bring a longer overall survival (OS) to the patients (HR: 0.530,95% CI: 0.456-0.616, p <.001) and progression-free survival (PFS) (HR: 0.740,95% CI: 0.649-0.844, p <.001), as well as higher objective response rates and disease control rates. And the result analysis also shows that the PNI value of 40–45 has a good predictive potential (10). Dai et al. studied the correlation between the preoperative PNI index level of ovarian cancer and the surgical prognosis. By analyzing 2050 patients with ovarian cancer who received surgical treatment, it was found that patients with low PNI had shorter OS and PFS than those with high PNI (HR = 1.82, 95% CI = 1.30-2.55, p <.05). It was proposed that low preoperative PNI is an adverse prognostic indicator for patients with ovarian cancer (27). Some articles have found that there is also an association between PNI and the prognosis of SCLC. Jiang et al. included a total of 9 studies involving 4,164 SCLC patients and found that low PNI led to SCLC OS (HR =1.43, 95% CI: 1.24-1.64, p <.001), but there was no significant correlation with PFS (28). Li et al. included 93 SCLC patients who received radiotherapy to explore the prognostic effect of PNI. The final results indicated that the OS of patients with PNI ≤47.7 was significantly shorter than that of patients with PNI >47.7, and PNI ≤47.7 could indicate a poor prognosis for patients with SCLC (29). Through the integrated analysis of the above literature, we can find that the PNI index can be used as an important prognostic indicator for clinical treatment evaluation in various types of cancers. Especially in the treatment of SCLC, PNI has a close connection with OS.

## 3 Immunotherapy using the prognostic nutritional index

A well-known aspect of cancer is its evasion of immune surveillance, particularly with the development of antibodies against the inhibitory immune checkpoint protein PD-1 or its ligand PD-L1 (30, 31). The PNI acts as a strong marker of overall immune health and nutritional condition, showing notable alterations during immune checkpoint blockade (ICB) treatment for SCLC. The elevation of PNI is mechanistically linked to enhanced tumor-infiltrating lymphocyte (TIL) intensity, particularly CD4^+^ and CD8^+^ T cells, which are critical for anti-tumor immune responses (32, 33). High PNI scores correlate with the inflamed SCLC-I molecular subtype, characterized by an immunologically active tumor microenvironment (TIME) with abundant TILs and elevated antigen presentation capacity. This may potentially augment the efficacy of ICB (32, 34). New therapeutic strategies have been developed thanks to improved anti-tumor immune responses and progress in molecular studies (23, 31).

Deng et al. reported that in extensive-stage SCLC, patients with high PNI exhibited significantly longer PFS and higher durable clinical benefit rates compared to those with low PNI (32). As a risk factor for poor prognosis of cancer patients, low PNI is of great significance for disease manifestation and clinical staging, which can be used to evaluate the prognosis of cancer patients (28, 35). Conversely, Tian et al. noted that PNI’s prognostic value is limited in SCLC patients receiving first-line chemotherapy alone, though its predictive capacity improves in specific contexts, suggesting context-dependent utility. The mechanism basis of elevated PNI may be related to the reduction of systemic inflammation and the improvement of nutritional status. By reducing the release of immunosuppressive cytokines and supporting lymphocyte proliferation, a favorable time is created for the ICB response (26, 32, 36).

The clinical progression of SCLC is difficult to predict. However, PNI calculated based on immune prognosis, has been shown to be related to the postoperative prognosis of various cancers, including LC (37). Some studies have shown that PNI can reflect the nutritional and immune status of patients with malignant tumors according to the ALB level and the TLC, while SCLC leads to the occurrence of hypoproteinemia, resulting in malnutrition, weight loss and poor prognosis, increasing the related death of such patients.

## 4 Immunotherapy for SCLC utilizing prognostic nutritional index

PD-1 is an important immunosuppressive molecule in the immunoglobulin superfamily. It was found in 1992 when T cell hybridoma cells died. However, PD-1 knockout studies confirmed that thymic T lymphocytes have no direct relationship with PD-1, and only serve as a regulator of T cell response (38–40). PD-1 forces T cells to ignore attacking cancer cells, but antibody-mediated PD-1 blocking function stimulates activated T cells to recognize the existence of cancer cells and release toxins (40). Sun et al. used immunohistochemistry to detect the surgical tissues of 102 patients with SCLC and proved that PD-1 and PD-L1 are key elements of representative potential markers of SCLC (41).

**Figure 1:**
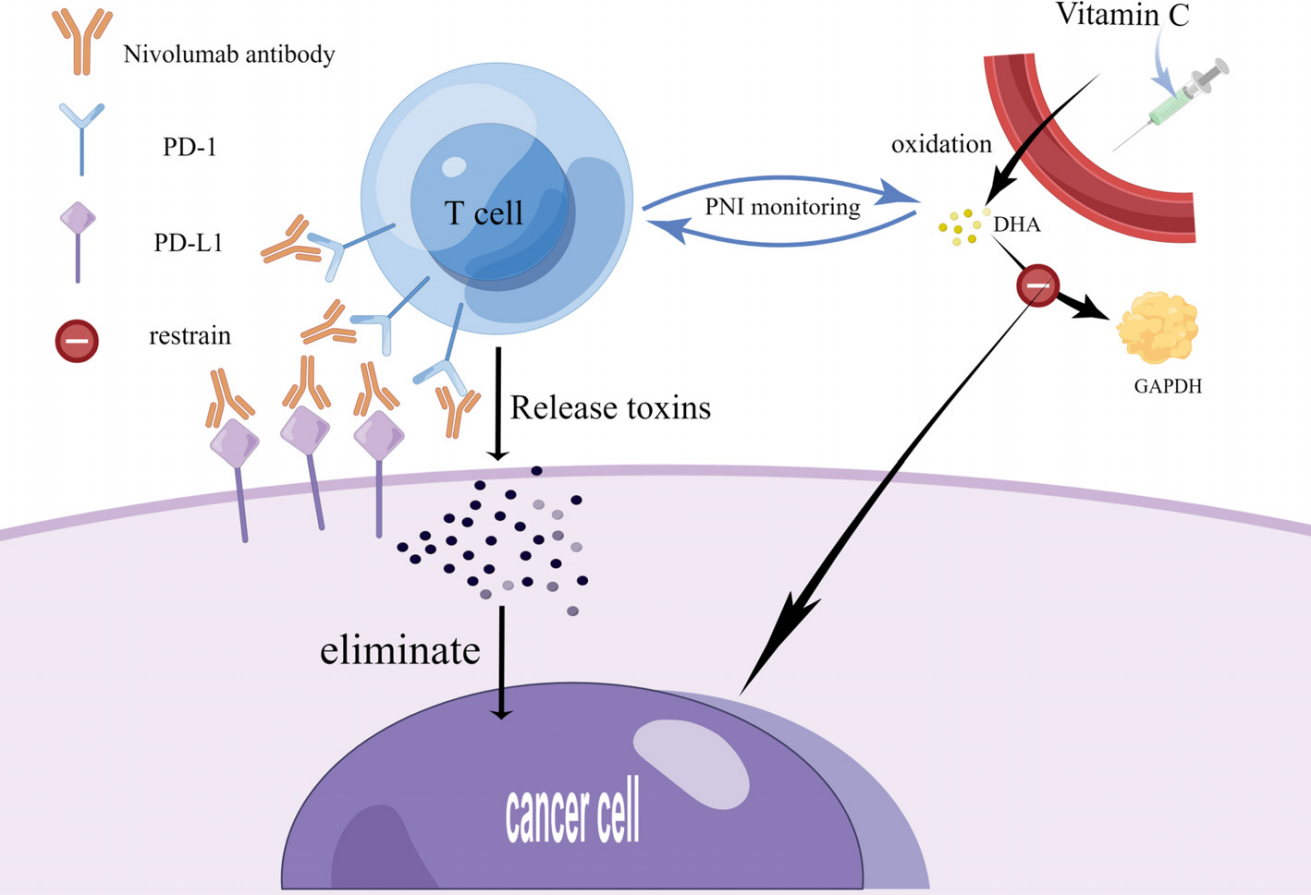
Current therapy strategies for small cell lung cancer. This figure was drawn by Figdraw. On the one hand, SCLC patients can take monoclonal antibodies (Nivolumab) to block the binding of PD-1 to PD-L1, which facilitates the activation and proliferation of T cells and causes them to release toxins to kill cancer cells, thus exerting an anti-cancer effect. On the other hand, SCLC patients can also exert an anti-cancer effect by injecting vitamin C, which undergoes oxidation in the body to produce DHA, and DHA inhibits the production of GAPDH. The dose of monoclonal antibodies and vitamin C can be regulated by indirectly monitoring the number of T cells through PNI. PD-1-programmed death receptor 1; PD-L1-programmed death receptor ligand 1; DHA- docosahexaenoic acid; GAPHD-glyceraldehyde-3-phosphate dehydrogenase.

PD-1 is referred to as an ‘immunosuppression checkpoint’. In the typical PD-1/PD-L1 pathway, PD-L1 on tumor cells and PD-1 on T cells interact to initiate inhibitory signals, reducing the T cell response. Meanwhile, these inhibitory signals are received by antibodies of PD-1 and its ligands, which alleviate T cell inhibition and block it by preventing the trans-action of PD-1 and PD-L1. It facilitates the activation and proliferation of T cells and causes them to release toxins to kill cancer cells, thus exerting an anti-cancer effect. In addition to the methods previously discussed for inhibiting cancer cells, anti-cancer effect can be realized by injecting vitamins and carotenoids. Of these, vitamin C is oxidized in the body to dehydroascorbic acid, which inhibits the production of glyceraldehyde 3-phosphate dehydrogenase and leads to anti-cancer effects. The dose of monoclonal antibodies and vitamin C can be regulated by indirectly monitoring the number of T cells through PNI (**Figure 1**) (17, 42). It has been shown that the PNI score can effectively predict the efficacy and prognosis of PD-L1 inhibitors combined with first-line chemotherapy in patients with extensive-stage SCLC. PNI involves blood markers that can be obtained by hematological tests, which have the advantages of simplicity, affordability, and less invasiveness.

Immunotherapy, especially PD-L1 inhibitors, has become a transformative strategy for SCLC. It provides moderate but significant survival benefits for some patients with advanced diseases. The mechanism of ICB in SCLC involves disrupting PD-1/PD-L1 interactions, thereby restoring T-cell-mediated tumor cytotoxicity and counteracting the immunosuppressive TIME characteristic of SCLC (26, 32). Horn et al. and Paz-Ares et al. have respectively reported the results of clinical trials, they demonstrated that the combination of atezolizumab and durvalumab with platinum-etoposide can prolong PFS and OS in the treatment of ES-SCLC. Although the remission rate was limited due to TIL infiltration and low PD-L1 expression (26, 36). The SCLC-I subtype of inflammation is rich in TILs and shows greater reactivity to ICB, emphasizing the key role of TIME in the treatment outcome (32, 34). Building on these observations, the elevation of the PNI in SCLC patients undergoing ICB therapy highlights its utility as a prognostic biomarker. According to Deng et al., a high PNI was linked to better PFS and greater CD8^+^ T-cell infiltration, indicating that PNI represents an enhanced systemic immune condition favorable for ICB effectiveness (32). In contrast, Tian et al. noted that PNI’s prognostic value is less pronounced in a chemotherapy-only setting, emphasizing its specific relevance in the immunotherapy context (33). By integrating the systemic immune status and local TME dynamics, PNI has become a practical and non-invasive biomarker for identifying SCLC patients who may benefit from ICB. The dynamic changes in PNI can better guide clinical strategies for personalized immunotherapy, thereby improving prognostic outcomes in SCLC (26, 32, 33, 36). Clinicians should consider this innovative biomarker when making clinical decisions, risk stratification management and selecting optimal patients for PD-L1 inhibitors. It is clear that PNI can be used as a risk indicator for real-time monitoring of immunotherapy in SCLC patients (32).

## 5 The role of dietary therapy based on PNI in the treatment of SCLC

Cancer associated cachexia is often underestimated in many cancer treatment processes. In the prognosis of cancer patients, the proportion of patients with advanced cancer presenting with cachexia symptoms is as high as 80% (43). It is defined as a multifactorial syndrome accompanied by persistent muscle mass loss and fat mass loss, which cannot be completely reversed by conventional nutritional support and is associated with a reduced survival rate (44). Chronic inflammation may be an important factor leading to cancer associated cachexia. ALB can be used to indicate the inflammatory condition and is a good indicator of the degree of cachexia (45). The reduction of ALB may cause a loss of appetite and even lead to anorexia (46). Another factor related to cachexia is impaired immune function. Lymphocytes can largely represent the relationship between the immune system and cancer (47). And as the severity of malnutrition increases, the absolute lymphocyte count will decrease (48). Through the analysis of the possible influencing factors of cancer associated cachexia, albumin and lymphocytes play an important role in the prognosis process of tumors (49). The calculation of the PNI index usually also includes two important indicators, albumin and lymphocytes. Therefore, PNI may be an important biomarker for showing the nutritional status in the prognosis process of cancer. It is reported that in SCLC, the albumin to globulin ratio and albumin level are related to the prognosis of patients (49). Therefore, we can calculate the serum protein level and the total lymphocyte count in peripheral blood through the prognostic indicator PNI of SCLC to reflect the nutritional and immune status of patients. (8, 17, 50–52).

The PNI status in a significantly larger cohort of surgically resected patients with SCLC demonstrated that a low PNI is linked to poorer clinical outcomes. Furthermore, considering the substantial influence that postoperative adjuvant chemotherapy (ACT) can have on the survival of patients with limited resected SCLC, the finding that PNI remained an independent prognostic biomarker irrespective of ACT enhances its potential for broader clinical application. Since PNI can be assessed by routine clinical trials, PNI can be a valuable prognostic indicator for SCLC patients (32, 53, 54).

Besides, we compare the etiology, pathogenesis, clinical manifestations, differential diagnosis, treatment and clinical prognosis of SCLC and non-small cell lung cancer (NSCLC) (**Table 1**).

**Table 1:** Comparison between SCLC and NSCLC.

Compare items	Small lung cancer	None-small lung cancer	References
Pathogenesis	The mutual activation of PD-1 and PD-L1 will change the metabolism of T cells, so as to participate in the suppression of immune response and maintain peripheral tolerance, and its overexpression can make tumor cells evade immune surveillance	Persistent hypoxia, cellular stress and EMT overexpression can lead to NSCLC	(55) (56)
differential diagnosis	PNI, a prognostic marker for SCLC, reflects the nutritional and immune status of patients by calculating serum protein levels and total lymphocyte counts in peripheral blood.	EMT signature is inversely associated with T-cell infiltration in NSCLC	(57) (8) (52)
competitive treatment	The combination of alkalization therapy and intravenous vitamin C treatment may be associated with favorable outcomes in patients with SCLC receiving chemotherapy.	Effectiveness of nutritional intervention combined with exercise in maintaining physical function and nutrition in patients with cachexia.	(50) (17)
prognosis	A variety of apoptotic agents, compounds that use DNA to repair defects, immunotherapeutic agents and antibody drug conjugates can effectively improve the poor prognosis of SCLC	BLACAT1 is an independent prognostic factor for survival in patients with non-small cell lung cancer	(58) (51)

## 6 Conclusion and prospects

PNI plays an important role as a measure of nutritional status in the treatment of SCLC. By integrating the relevant literatures in recent years, we found that the traditional radiotherapy and chemotherapy methods for SCLC have strong toxicity to patients. Immunotherapy for SCLC based on PNI may be able to reduce the impact on patients. And it was found that PNI is widely used in the clinical application of cancer treatment and has strong applicability. This paper demonstrates the importance of PNI by elaborating it in the role of SCLC immunotherapy. Furthermore, we also propose the combination of dietary nutrient intake and PNI scores as a new treatment method. We believe that changing diet may become another major new direction to change the treatment of SCLC. Studies have shown that carotenoids, vitamin D and vitamin E can help prevent the occurrence and development of SCLC, and injecting vitamin C may also play a role (59, 60).

In clinical research, experts have regarded drug treatment as the main breakthrough of SCLC, but the lack of biomarkers has become a major challenge in the early diagnosis and treatment of it. According to PNI, we can correct the malnutrition of SCLC patients by improving their diet. And by combining first-line drugs and diet treatment, we hope to effectively treat SCLC patients from a new perspective of PNI, so as to reduce their pain. In conclusion, PNI will become a new strategy for comprehensive immunotherapy of SCLC.
